# Effects of *Melissa officinalis* Phytosome on Sleep Quality: Results of a Prospective, Double-Blind, Placebo-Controlled, and Cross-Over Study

**DOI:** 10.3390/nu16234199

**Published:** 2024-12-04

**Authors:** Francesco Di Pierro, Davide Sisti, Marco Rocchi, Annalisa Belli, Alexander Bertuccioli, Massimiliano Cazzaniga, Chiara Maria Palazzi, Maria Laura Tanda, Nicola Zerbinati

**Affiliations:** 1 Department of Medicine and Technological Innovation, University of Insubria, 21100 Varese, Italy; f.dipierro@vellejaresearch.com (F.D.P.); nicola.zerbinati@uninsubria.it (N.Z.); 2 Microbiota International Clinical Society, 10123 Torino, Italy; alexander.bertuccioli@uniurb.it (A.B.); maxcazzaniga66@gmail.com (M.C.); 3 Scientific & Research Department, Velleja Research, 20125 Milano, Italy; 4 Department of Biomolecular Sciences, University of Urbino Carlo Bo, 61029 Urbino, Italy; davide.sisti@uniurb.it (D.S.); marco.rocchi@uniurb.it (M.R.); 5 Department of Medicine and Surgery, University of Insubria, 21100 Varese, Italy; marialaura.tanda@uninsubria.it

**Keywords:** rosmarinic acid, hydroxycinnamic acids, oral bioavailability, GABA-T

## Abstract

Background: *Melissa officinalis* standardised extracts, characterised by the presence of hydroxycinnamic acids, have been experimentally demonstrated to be endowed with anti-anxiety and anti-insomnia pharmacological actions. These effects, probably attributable, at least in part, to the role played by rosmarinic acid on GABA-T, have not always been observed in a reproducible manner in humans, perhaps due to the poor bioavailability of these compounds. Methods: as nutraceuticals and botanicals could be an alternative option to prescription medications for alleviating symptoms of mild anxiety and insomnia, we have verified in a prospective, double-blind, placebo-controlled, and cross-over study the supporting role on sleep quality played by a *Melissa officinalis* highly standardised extract, formulated as Phytosome™ (MOP) to improve the oral bioavailability of its active polyphenolic components. Results: results showed a significant reduction in the ISI score in the treated group, with an average of 6.8 ± 4.1 compared to 9.7 ± 3.7 in the placebo group, indicating a significant reduction of 2.9 points (*p* = 0.003). The SWS phase duration increased by an average of 15%, while the REM phase decreased by 10%. Additionally, 87% of participants in the treated group reported improved sleep quality, compared to 30% in the placebo group, with significant differences measured by chi-square test (*χ^2^*_(4)_ = 21.01, *p* = 0.0003), highlighting the effects due to *Melissa officinalis* L. No significant changes in physical activity or anxiety levels were observed. Conclusions: these findings suggest that MOP may represent a natural and safe alternative to traditional pharmacological treatments for insomnia.

## 1. Introduction

*Melissa officinalis* (MO) is a spermatophyte plant belonging to the order Lamiales, family Lamiaceae, also known by the names “lemon balm”, “common balm”, “melissa balm”, and “sweet balm”. Opinions on the origin area of this plant are varied, but it is thought to have developed in the Mediterranean basin or the East Asian area. Regardless of its origin, it is now widely cultivated around the world, especially in temperate and subtropical climates [1]. MO has been known since ancient times, with a history spanning 10,000 years, particularly in traditional Persian medicine, which attributes to MO a calming activity and a tonic effect on the nervous system [2]. Modern medical uses of MO have been recorded mostly in European countries, the Mediterranean region, and Middle Eastern countries where clinical validation of MO has been provided for anxiolytic, antiviral, antispasmodic, mood-modulating, pro-cognition, and pro-memory effects [3,4]. Phytochemical investigations have revealed that this plant contains volatile compounds, triterpenoids, phenolic acids, and flavonoids. Crude extracts and pure compounds isolated from MO have exhibited numerous pharmacological effects, including interaction with acetylcholine-esterase, gamma amino butyric acid (GABA) receptors, and matrix metallo-proteinase 2 (MMP2), determining also a beneficial clinical effect on anxiety-related insomnia [3,4,5]. Hydroxycinnamic acids are thought to represent one of the most important classes of compounds characterising MO [5,6]. The class includes cinnamic acid, coumarin acids, ferulic acids, chlorogenic acid, and, mostly, rosmarinic acid [7,8]. Besides MO, rosmarinic acid, a naturally occurring esterification product of caffeic acid and 3,4-dihydroxyphenyl lactic acid, is one of the main natural polyphenols in spearmint (*Mentha spicata*), shiso (*Perilla frutescens*), and rosemary (*Rosmarinus officinalis*) [9]. Despite in vitro tests having clearly shown the activity of rosmarinic acid in inhibiting GABA-transaminase [10] and some animals studies having demonstrated its effect in decreasing sleep latency and increasing total sleep time [11,12,13], placebo-controlled human studies have shown controversial results on sleep quality [14,15]. The lack of a clear effect in humans could be due to its poor oral bioavailability [8,16,17,18]. Sleep is a finely regulated biological condition, and we can easily deduce that good sleep quality reflects an improvement in daytime quality of life. Sleep plays an essential role in recovery and energy conservation, and it is indispensable for life [19]. The problem of insomnia is often overshadowed by other issues such as mood disorders and anxiety, even though these phenomena are closely connected [20]. Insomnia is frequently associated with other sleep disorders, such as obstructive sleep apnea, periodic limb movements, respiratory disorders, narcolepsy, and idiopathic hypersomnia, but delayed sleep phase syndrome is a particularly common cause of insomnia. In individuals affected by this disorder, sleep is delayed relative to the biological clock, resulting in difficulty falling asleep and waking up at a scheduled time in the morning, leading to daytime problems such as poor concentration, sleepiness, and loss of interest [20,21]. Additionally, altered sleep duration and quality also produce significant effects on metabolic processes such as reduced glucose tolerance, lower levels of ghrelin (orexigenic hormone), and increased cravings for high-calorie foods, resulting in a higher risk of obesity [22]. Finally, upon analyzing a sample of 92,340 subjects, a linear progression linking sleep quality with the absence of disease and life expectancy was clearly observed, and indeed experimental sleep loss can cause death in animals by devastating gut oxidative effects that can be halted by the administration of antioxidants [23,24]. Since the primary function of sleep is to enable wakefulness, sleep quality could reflect the ability to efficiently perform daytime activities, as well as cognitive abilities and mood being strongly influenced [22]. However, it is challenging to provide an objective definition of “sleep quality” because there are no universal criteria for defining it, as it is rather subjectively perceived. It can be stated that sleep quality is generally defined by sleep continuity (e.g., sleep onset, sleep maintenance, and the number of awakenings) and the impact of sleep on daytime activities (e.g., feeling rested upon waking and throughout the day) [25]. Based on this definition of sleep quality, the ISI (Insomnia Severity Index) was chosen to evaluate perceived sleep quality. Currently, there are efforts to establish universal criteria for defining the concept of sleep quality, and indicators such as REM (rapid eye movement) and SWS (slow-wave sleep or “deep sleep”) phases are increasingly being considered [26]. Although some studies with antidepressant medications that induce a reduction in REM sleep do not show any significant deterioration in cognitive abilities, other studies attribute great importance to the REM phase for maintaining mental health [26,27]. SWS appears to be one of the components of sleep responsible for satisfying homeostatic needs, promoting good cognitive performance during the day [26]. Despite contrasting opinions, SWS seems to be a key component for the consolidation of learning material from the previous wakefulness period and the restoration of synaptic homeostasis [28]. However, REM and SWS complement each other, and a balanced cycle between REM and SWS is necessary for individual health. Slow-wave sleep has been extensively studied as a key predictor of sleep quality, being considered the most restorative sleep stage. Keklund and Akerstedt have stated that the strongest predictors of sleep quality are indeed SWS and sleep efficiency [29]. Some evidence has also shown that SWS can be improved through various techniques, such as progressive muscle relaxation (PMR), which appears to significantly increase SWS while leading to a significant reduction in REM sleep, as well as listening to slow-wave sleep brain-wave music before bedtime [30,31]. Recent studies showed that SWS is particularly important in individuals with Alzheimer’s disease (AD), as it is directly linked to learning and memory consolidation processes. Individuals with AD report a shorter duration of SWS, which is associated with the severity of cognitive decline [32]. Not only do individuals with AD spend less time in SWS, but a reduction in SWS also significantly increases the accumulation of cerebral beta-amyloid plaques, with sleep alterations occurring even in the preclinical phase of AD [33]. Given the importance of a correct distribution of sleep phases for achieving the best possible sleep quality, an increase in time spent in the SWS phase might reflect an improvement in perceived sleep quality. As nutraceuticals and botanicals could be an alternative option to prescription medications for alleviating symptoms of mild anxiety and insomnia, we have verified the supporting role related to sleep quality played by an MO extract, highly standardised in hydroxycinnamic acids and containing rosmarinic acid, formulated as Phytosome™ (MOP) to improve the oral bioavailability of its active polyphenolic components [34,35,36].

## 2. Materials and Methods

### 2.1. The Study

The study was a prospective, double-blind, placebo-controlled, cross-over, and no-profit study. It was conducted according to the principles stated in the Declaration of Helsinki, and it was approved by the Ethics Committee for Human Experimentation of Urbino University Carlo Bo (Approbation nr. 72 of 27 July 2023) and was registered at www.clinicaltrials.gov with identification number NCT05950932.

After informing the participants about the purposes and methods of the study, informed consent was obtained from each participant. Thirty participants (13 males and 17 females) were recruited from two different clinical centres, one located in central Italy (Pesaro) and the other in northern Italy (Milan). The inclusion criteria were an age range of 18 to 65 years and perception of fatigue upon waking and unrefreshing sleep, while subjects meeting any exclusion criteria were disqualified. The exclusion criteria included conditions such as pregnancy, breastfeeding, or intention to become pregnant during the course of the study; consumption of anxiolytics, antidepressants, or hypnotics 15 days before the start of the study; diabetes; asthma; thyroid dysfunctions (hypo- or hyperthyroidism); alcoholism; smoking; and current or recent past use of drugs or other herbal remedies for sleep disorders. Participants were informed about the use of a Garmin VenuSq wrist-worn device (Garmin Ltd., Olathe, KS, USA), and each was assigned a personal email, differing only by a serial number, and a password to access the Garmin account on a smartphone application. Each participant was then given a kit containing two boxes (one with Melissa Phytosome^®^; Pharmextracta S.p.A., Pontenure, Italy; 00 mg/capsule, the other with a placebo) identical in appearance, size, shape, and colour of the tablets inside. Following the assignment of subjects to the two experimental groups, both boxes in each kit were previously numbered with “1” or “2”, numbers corresponding to the order of intake. To determine which subject should belong to one of the two groups (Group 1: MOP first, placebo after; Group 2: placebo first, MOP after) and to ensure homogeneity between the two experimental arms, a block randomisation method (block dimension = 4) was used for the 30 participants.

### 2.2. The Products

The product under evaluation (Meloff^®^, Pharmextracta SpA, Pontenure, Italy) was registered as a dietary supplement in compliance with Italian law no. 169/2004 (registration number: 161797). Each tablet, lactose- and gluten-free, contained 200 mg of active *Melissa officinalis* Phytosome^®^ (MOP, Relissa™, Indena S.p.A, Milano, Italy). The placebo tablets, produced by the same contract manufacturer (S.I.I.T., Trezzano S/N, Milan, Italy) who manufactured the tested product, were prepared to match the active product in form, colour, consistency, dissolution time, and flavor, but they did not contain MOP.

### 2.3. Outcomes and Tools to Measures

The main outcome of the study was to evaluate the action of MOP in sleep quality. This target was supposed to be obtained by evaluating (i) total sleep duration, (ii) light sleep, (iii) SWS, and (iv) REM phase and distribution of sleep stages. These were measured using a Garmin VenuSq wrist-worn device (Garmin Ltd. USA), which can monitor heart rate and blood oxygen saturation levels in peripheral vessels with Pulse Ox technology, as well as a standard accelerometer [37]. All sensors remain active throughout the entire time the watch is worn, allowing the device to provide continuous feedback. The Garmin VenuSq has been validated to measure steps, distance, energy expenditure, heart rate, speed, elevation, and sleep and has been used in several studies to evaluate the effects of different physical activity interventions on the sleep of pregnant women or to evaluate the role of wearable devices in promoting physical activity [38,39,40,41].

Changes in sleep quality were also assessed using the ISI questionnaire, consisting of 5 items (the first of which is further divided into 3 items, with 7 items total) regarding the self-reported severity of insomnia problems, satisfaction with current sleep, the extent to which sleep interferes with daytime functioning, the extent to which the sleep problem is noticeable to others, and the current concern/stress caused by sleep problems. Based on the responses, a score is assigned, and the subject is placed into one of the following 4 categories: absence of clinically significant insomnia; subthreshold insomnia; clinical insomnia of moderate severity; and severe clinical insomnia. The higher the score, the greater the degree of insomnia detected [42]. Additionally, the IPAQ (International Physical Activity Questionnaire) [43] and the STAI-Y (State-Trait Anxiety Inventory, Form Y) [44] were used. The IPAQ is a questionnaire used to measure in METs (metabolic equivalent of tasks) the level of physical activity of people, considering various aspects such as vigorous physical activity, moderate physical activity, and time spent sitting. The STAI-Y is a questionnaire used to measure levels of state anxiety (temporary anxiety) and trait anxiety (anxiety as a stable personality characteristic). Secondary outcomes were tolerability, compliance, and appearance of side effects.

### 2.4. Study Design

**Figure 1:**
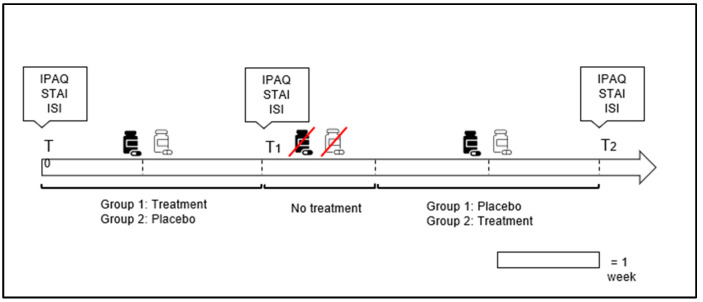
Graphical illustration of temporal phases of intervention, in which the timing of administration for the International Physical Activity Questionnaire (IPAQ), State-Trait Anxiety Inventory (STAI), and Insomnia Severity Index (ISI) is outlined.

To assess the effects of MOP, Garmin VenuSq was worn continuously, day and night, by all subjects, and questionnaires were administered at three different times: T0, T1, and T2. Specifically, T0 corresponds to the day of or before the start of the supplement intake, T1 corresponds to the day following the end of supplementation 1 (either MOP or placebo), and T2 was administered at the end of supplementation 2 (either MOP or a placebo). Additionally, as shown in Figure 1, there was a 7-day washout period between the end of supplementation 1 (two weeks) and the start of supplementation 2 (two weeks). With regard to supplementation, all subjects took 2 tablets of MOP or a placebo every evening 30 min before going to bed. To ensure daily intake, an evening reminder was sent to each participant throughout the study. The participants and the evaluators recruiting the participants were blinded to the supplementation conditions.

### 2.5. Statistical Analysis

**Figure 2:**
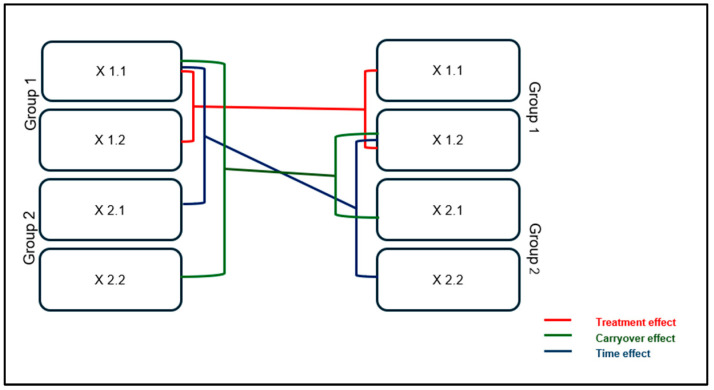
Graphical representation of the cross-over design in which “Group 1” represents the group that took MOP during the first two weeks and a placebo during the last two, while “Group 2” performed the opposite.

The sample size was calculated to minimise Type I error (α), which was set at 0.05, while Type II error (β) was set at 0.10, resulting in a power of 90%. With a 1:1 allocation ratio, a cross-over analysis requires a total of 30 participants to be included in this two-group study if the actual difference between supplements is at least one hour, with a standard deviation of 1.56. The probability of detecting a supplementation difference at a two-tailed significance level of 0.05 is 80% (1-β). The questionnaire scores have been analysed by a multivariate GLM (general linear model) for repeated measures. The analyses of participant outcomes have been conducted based on the intention-to-treat (ITT) principle. In a simple two-period, two-group cross-over study (Figure 2), each average effect was reported as “Xm.n”, where m (1 or 2) represents the supplement (MOP or placebo), and n (1 or 2) denotes the period (1 = from day 1 to day 15; 2 = from day 21 to day 35). The period from day 16 to day 20 was considered a washout, to avoid the influence of carryover effects from supplementation 1. To test the supplement effect, the sum of the results from supplementation 1 was compared with the results from supplementation 2 (x1.1 + x1.2 versus x2.1 + x2.2). To study the effect of time, the sum of results in period 1 was compared with the results in period 2 (x1.1 + x2.1 versus x1.2 + x2.2). Finally, to assess the carryover effect, the sum of results in group 1 was compared with the results in group 2 (x1.1 + x2.2 versus x1.2 + x2.1). These null hypotheses are tested using paired *t*-tests with appropriate post hoc corrections to control for Type I error. Data analyses were performed using Excel SPSS (version 22) and software R studio (version 2024.09.1), while the graphs were created with GraphPad Prism 8.0.

## 3. Results

### 3.1. Participants

**Figure 3:**
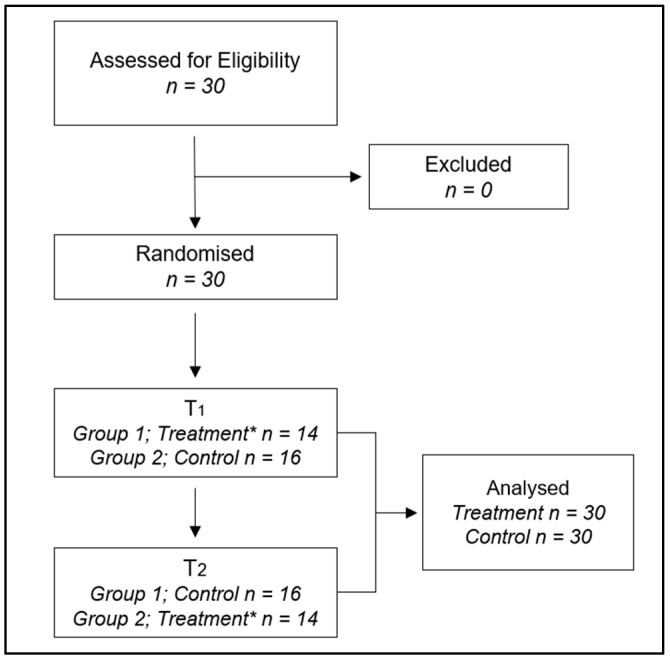
Flowchart of study participants. *: Treatment with MOP.

All 30 eligible subjects (features are shown in Table 1) who participated in the trial completed the study over a one-month period (from March to April) in 2024. The flow diagram is shown in Figure 3. Differences were identified using the chi-square test for the distribution of gender in the sample and education levels, while a *t*-test was employed to assess differences in age, weight, height, and BMI. In this study, both height and weight were found to be statistically different between males and females, while BMI (body mass index) did not show significant differences. This result can be understood in light of the way BMI is calculated and how the differences in height and weight interact [45]. Since BMI is a relative measure that standardises weight in relation to height, the differences in absolute terms between males and females may not translate into differences in BMI. The variability within each group (males and females) in terms of height and weight may also contribute to the non-significant BMI difference. No side effects related to MOP supplementation were detected during the study.

### 3.2. Physical Activity

**Figure 4:**
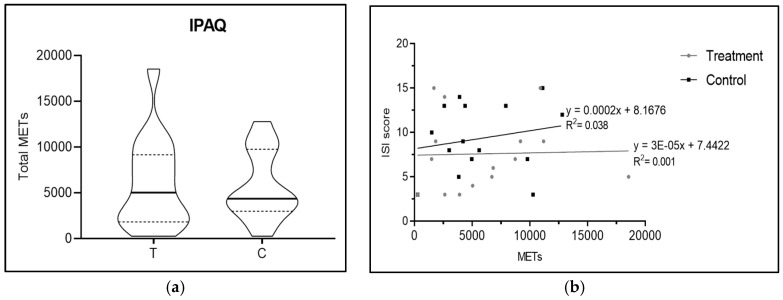
(**a**) shows a violin plot of International Physical Activity Questionnaire score, for which there are no significant differences between the two groups (*p* = 0.63); (**b**) shows linear regression lines regarding METs and ISI score.

As shown in the Figure 4a,b, there were no significant differences in physical activity, with *p* = 0.63 (measured by the difference in IPAQ scores between the MOP-supplemented and the placebo group), highlighting that physical activity levels between the two groups were similar, thereby eliminating the potential confounding factor that increased physical activity might facilitate sleep. The median (Q1–Q3) for the MOP group was found to be 3060 METs (1462.5–6956.2), while in the placebo group the median (Q1–Q3) was 3870 METs (2127.5–6525). This is further emphasised by the linear regression lines (Figure 4b) regarding the METs–ISI score relationship in the supplement and placebo groups: although the two lines may appear graphically different, there is no statistically significant difference.

### 3.3. Anxiety State

**Figure 5:**
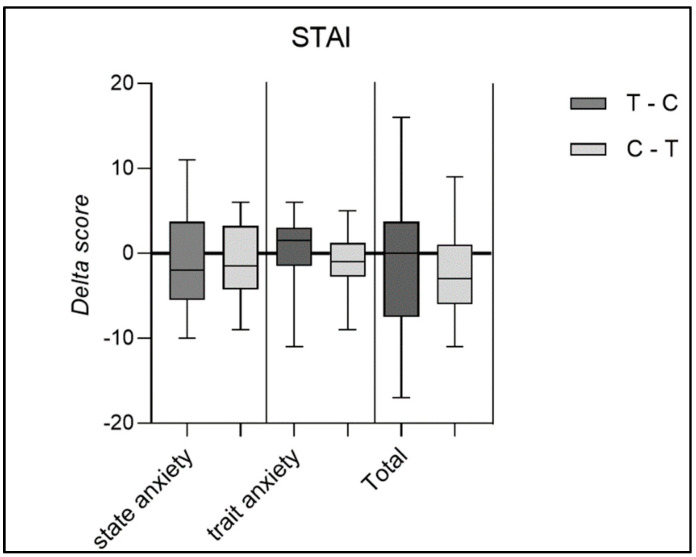
Delta score of State-Trait Anxiety Inventory questionnaire considering state anxiety, trait anxiety, and the total score in the two groups.

Similarly, for the STAI results, (Figure 5), no statistically significant differences were found between the pre- and post-supplementation periods, highlighting that the improvement in sleep quality was not influenced by a reduction in either state anxiety or trait anxiety.

### 3.4. Insomnia Severity Index

**Figure 6:**
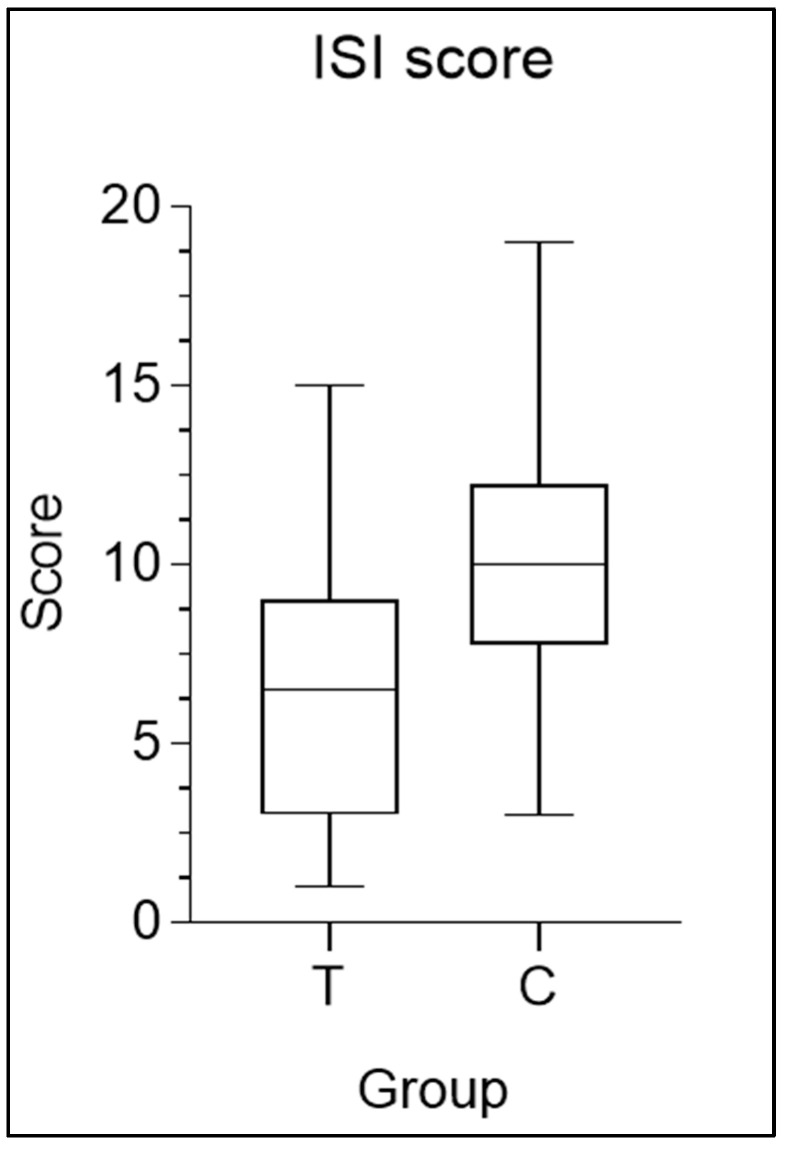
Box-and-whisker plot of mean scores of Insomnia Severity Index questionnaire, in which “Group 1” represents the group that took MOP during the first two weeks and a placebo during the last two, while “Group 2” performed the opposite.

**Figure 7:**
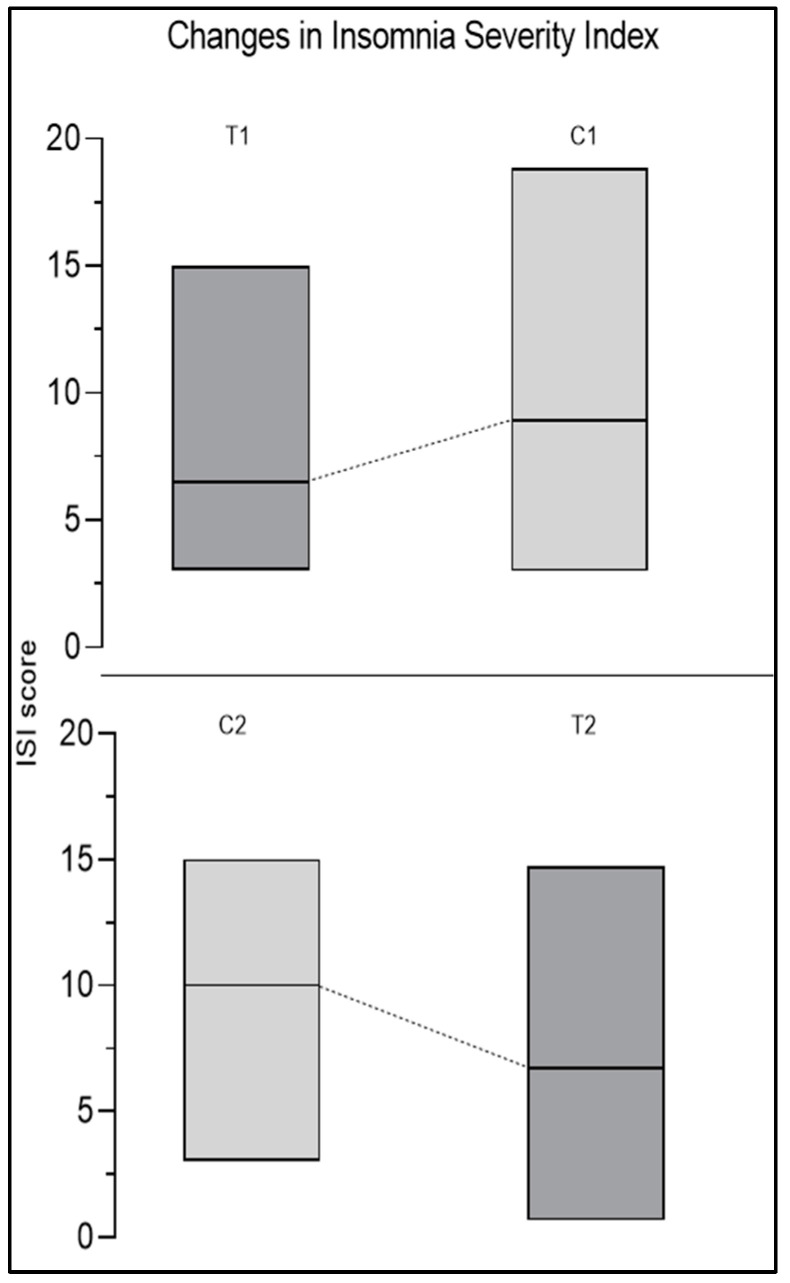
Changes in Insomnia Severity Index scores. It can be observed that there is an increase in the ISI score from treatment (T1) to placebo (C2). Conversely, in the lower part of the graph, it can be noted that in Group 2, the opposite occurred, with a decrease in the ISI score from the placebo period (C2) to the treatment period (T2).

The most interesting results are evident from a decrease in the ISI score, a significant increase in deep sleep, and a significant decrease in the REM sleep phase in the MOP-supplemented group. At time T0, before the start of supplementation, 26.7% reported no insomnia, 60.0% reported subclinical insomnia, and 13.3% reported moderate insomnia. Figure 6 shows the average ISI scores for the MOP and placebo groups. The group (placebo) had an average score of 9.7 ± 3.7, while the supplement group had an average score of 6.8 ± 4.1, highlighting a significant average reduction of 2.9 points (*p* = 0.003). Since the ISI score provides information on the severity of insomnia, with higher scores indicating more severe insomnia, a reduction in the score indicates an improvement in the condition. The change in the ISI score can also be observed in Figure 7. The upper part of the graph of Figure 7, shows the trend of the ISI score in the group that first took MOP and then the placebo. It is immediately noticeable that the score increased after the discontinuation of the supplement, resulting in a worsening of insomnia severity. In contrast, the lower part of the graph of Figure 7, which represents the group that took the MOP first and then the placebo, shows the opposite trend (a reduction in the ISI score), demonstrating that the intake of the supplement led to an improvement in insomnia condition among the participants.

### 3.5. Sleep Quality Parameters

**Figure 8:**
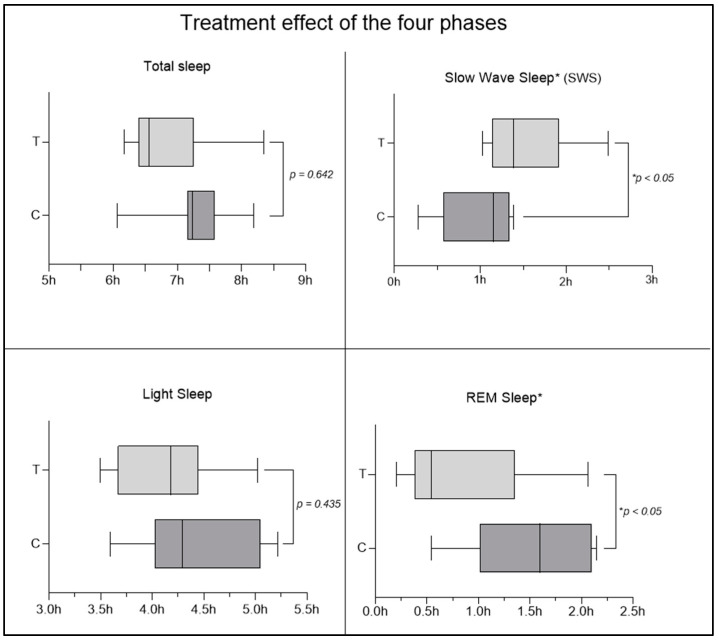
Treatment effect of the four phases of sleep. Although no significant changes were observed in total sleep time and light sleep phase, significant changes were detected in SWS and REM sleep.

**Figure 9:**
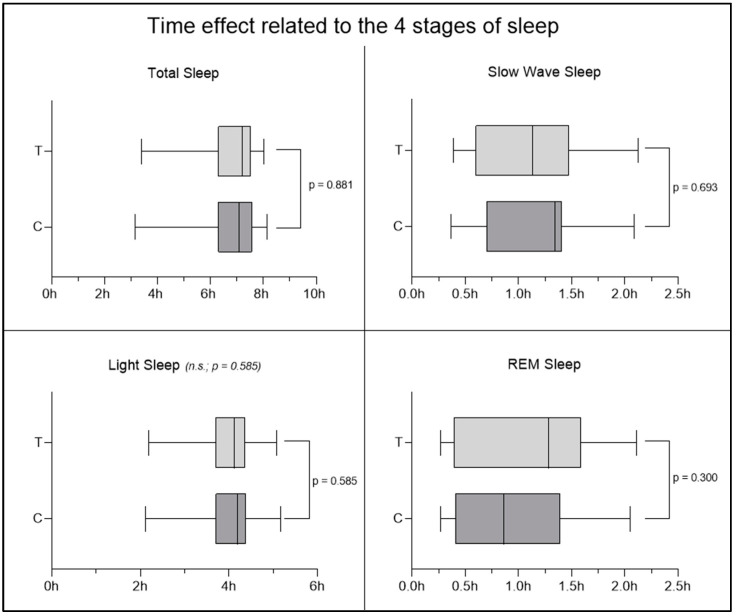
Time effect related to the four stages of sleep. No significant results were observed regarding the time effect, indicating no influence of the time factor on the interpretation of the results.

**Figure 10:**
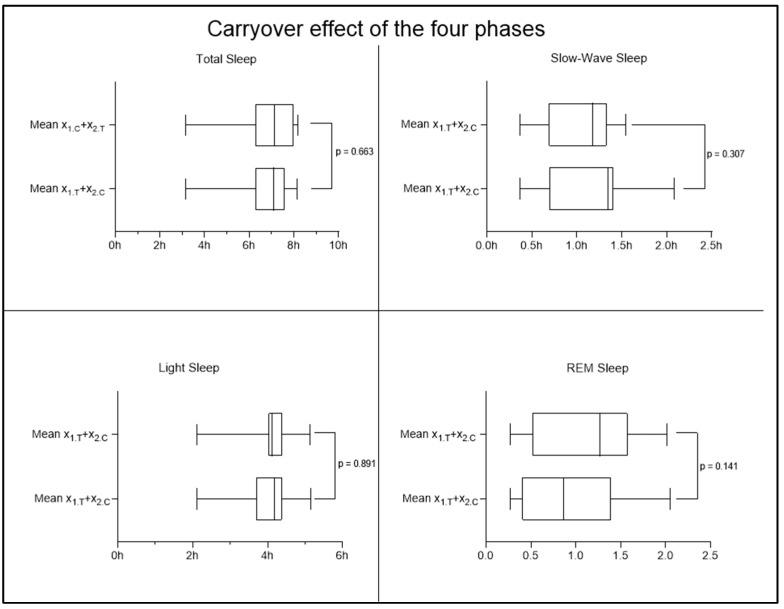
No significant results were observed regarding the carryover effect in any sleep phase. On the y-axis, the average time spent in each phase is shown, to be interpreted as follows: e.g., for X1.T: 1 = group considered; T = two weeks of treatment with MOP considered.

An interesting result was observed regarding the improvement in sleep quality parameters. Cross-over analysis revealed statistically significant differences (*p* < 0.05) in the deep sleep and REM sleep phases, with an increase in the time spent in deep phase and a decrease in the time spent in the REM phase in the MOP group (Figure 8), suggesting an improvement in sleep quality. No significant changes were observed in light sleep time, wake time, and total sleep time. To ensure that the observed results are due to the supplement and not to other external or prior factors, the effects of time and carryover were also investigated. In the case of the time effect, the non-significance obtained from the analysis of the four sleep phases (Figure 9) allows us to exclude the possibility that the improvement (or worsening) of participants’ conditions is due to the mere passage of time, regardless of the supplement received. The results obtained, therefore, allow us to affirm that the observed changes in the results are not attributable to the passage of time but to MOP supplementation. To also rule out the possible persistence of the previous supplementation and its potential influence at the beginning of the second period, the carryover effect was investigated, which did not show significant changes (Figure 10). This allows us to confirm that the effect of the first period was completely eliminated thanks to the one-week washout period between the end of supplementation 1 (MOP or placebo) and the start of supplementation 2 (placebo or MOP). These findings provide further evidence of the robustness of the study, ensuring control over these potential confounding factors.

### 3.6. Subjective Perception of Sleep Quality

**Figure 11:**
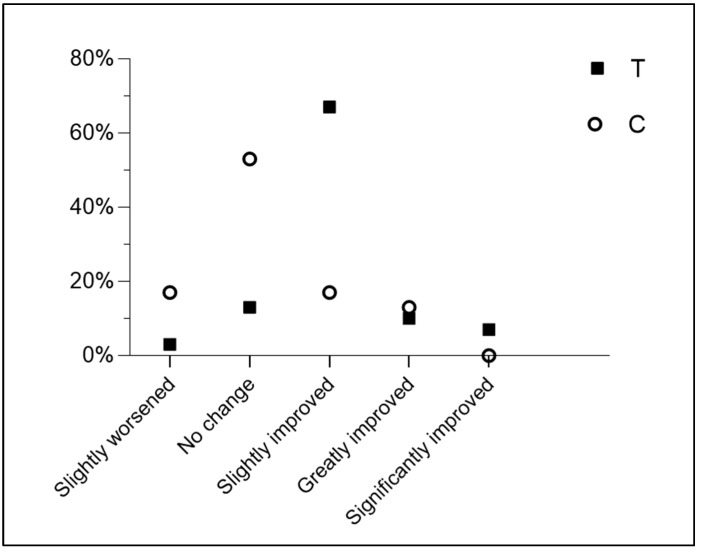
Subjective improvements in sleep quality expressed as a percentage.

Another result concerns the subjective perception of improved sleep quality. While 87% of the subjects reported varying degrees of improvement (slightly improved, greatly improved, significantly improved) during the period of taking the *Melissa officinalis* L. supplement in phytosome form, only 30% of the placebo group reported perceived improvements (likely due to the placebo effect itself), and none of them reported the highest level of improvement. These results are shown in Table 2 and Figure 11. A chi-squared test was performed to evaluate the differences in response distribution between groups T and C across categories. The results indicated a statistically significant difference in the distribution of responses between the two groups (*χ*^2^_(4)_ = 21.01, *p* = 0.0003). The expected frequencies were [10, 10] for “no change”, [3, 3] for “slightly worsened”, [12.5, 12.5] for “slightly improved”, [3.5, 3.5] for “greatly improved”, and [1, 1] for “significantly improved”. These findings suggest that the pattern of responses differs significantly between the groups.

## 4. Discussion

Insomnia is the most common sleep disorder and one of the most prevalent pathological conditions in primary care. In a study conducted in 2022 involving 748 Italian subjects over 50 years of age, a high use of medications, including off-label drugs, was reported to address insomnia, whether diagnosed or not [46]. Given the evidence of the greater efficacy of the phytosome forms [47,48] for certain plant-based active ingredients and the concurrent excessive use of medications to treat insomnia conditions, we aimed to develop a nutraceutical formulation of *Melissa officinalis* Phytosome™ (MOP) to improve sleep quality and duration.

The study did not reveal significant changes in either the levels of physical activity performed or the perceived anxiety levels, as assessed respectively by the IPAQ and STAI-Y questionnaire. These results provide an excellent starting point for ruling out potential confounding factors. Several studies have shown that sleep quality is influenced by physical activity [49,50]. It has been demonstrated that individuals who engage in physical activity sleep better and longer than sedentary individuals, with greater effects if moderate or intense physical activity is performed and no effect in the case of light activities [49]. Since the participants in the study did not show significant changes in physical activity levels (expressed in METs), this potential confounding factor was ruled out. However, the benefits of physical activity on sleep may take longer to manifest or require more intensive interventions to achieve noticeable improvements.

In addition to physical activity, sleep quality is also influenced by anxiety, although the mechanisms are still not entirely understood [51]. Proserpio et al. also addressed identifying the main comorbidities of insomnia, with anxiety-depressive disorder ranking first, followed by other psychiatric disorders, cardiovascular diseases, and dementia [46]. Individuals with anxiety appear to experience effects on both the quality and duration of their sleep. Total sleep shows only a slight reduction in anxious individuals, while sleep continuity markedly decreases and is associated with a reduction in positive effects and a decrease in attention to positive stimuli [52,53]. As we have just described, anxiety and depression are often associated with chronic sleep disturbances, as they can lead to hyperarousal, difficulty relaxing, and negative thought patterns that interfere with falling and staying asleep [51]. Even without immediate changes in anxiety levels, persistent symptoms over time can exacerbate sleep issues. Although further studies are needed, deep sleep does not seem to decrease significantly in anxious individuals [52]. It is difficult to establish whether anxiety leads to sleep problems or whether sleep problems contribute causally to anxiety-related disorders. Due to the potential influence of anxiety on sleep quality and duration, we administered the STAI-Y questionnaire to assess state, trait, and total anxiety at the three different time points of the study. No significant changes were observed. This result allows us to exclude another potential confounding factor, namely the presence of anxiety, further emphasising that changes in sleep quality and quantity are not influenced by changes in participants’ anxiety levels.

To assess the subjective perception of sleep quality, the ISI was used [42]. Although several questionnaires for assessing insomnia exist in the literature, such as the Pittsburgh Sleep Quality Index [54], the Insomnia Symptom Questionnaire [55], and the Athens Insomnia Scale [56], the Insomnia Severity Index was preferred, as it is considered the easiest to complete and is deemed psychometrically valid for both screening purposes and for evaluating outcomes in both groups, given the abundant literature on the subject [57,58]. At time T0, before the start of supplementation, 26.7% of participantsreported no insomnia, 60.0% reported subclinical insomnia, and 13.3% reported moderate insomnia. After statistical analyses were conducted using a paired-sample *t*-test, statistically significant differences were found (with *p* = 0.003) between the MOP group and the placebo group, showing an average reduction of 2.9 points. The ISI score decreased when subjects transitioned from the placebo group to the MOP group, while it increased when considering the transition from MOP group to the placebo group. These results clearly indicate that both sleep quality and the subjective perception of sleep improved significantly following the administration of MOP compared to the placebo. The results confirm the properties of MOP in improving sleep quality [59].

In this study, a statistically significant increase in SWS was observed when subjects took MOP. Although there are discrepancies in the literature regarding the actual roles of SWS and REM sleep, a 2022 scoping review found a moderate correlation with improved daytime performance, motor speed, and executive function, as well as better perceived sleep quality and a sense of nighttime restfulness, suggesting that SWS may be crucial for sleep quality and optimal daytime performance [60]. Other studies show that slow-wave sleep is key to the consolidation of hippocampus-dependent declarative memory: this consolidation process is orchestrated by slow oscillations in the electroencephalogram and involves the reactivation of newly encoded representations, redistributing them to long-term storage sites in the hippocampus and neocortex [61]. Deep sleep is also implicated in the onset of dementia in older age. A 2023 study with a 17-year follow-up period aimed to determine whether SWS loss due to ageing could be associated with dementia risk, also examining whether the genetic risk of Alzheimer’s disease was linked to SWS loss [62]. The results obtained through Cox regression adjusted for various variables showed that each percentage point of annual SWS reduction was associated with a 27% increased risk of dementia [62]. As mentioned, SWS is most effective in memory consolidation, even compared to consolidation during wakefulness [63], as it is supported by spontaneous hippocampal replay, responsible for memory formation in the neocortex. The increase in daytime functionality, better perceived restfulness, and enhanced sense of nighttime restfulness associated with the increase in SWS were also observed in our sample following the intake of *Melissa officinalis* in phytosome form, in line with the decrease in ISI scores. However, we do not have enough information to claim an actual improvement in memory function.

REM sleep has been, and still is, the subject of numerous studies, particularly for its association with vivid dreams in humans. However, the functional physiology of the REM phase has been less studied than all other sleep phases [64]. REM sleep also appears to be associated with the nocturnal cycle of body temperature [65], and reduced REM latency has been shown to be one of the most robust and specific characteristics of sleep in depressed patients, being linked to an increased risk of depression [66]. It also appears that dysfunctions in REM sleep might have significant implications for cognitive decline and AD [67]. REM sleep also appears to play a role in mood regulation. In fact, it has been observed that early negative dreams (occurring at the start of sleep) reflect a process of mood regulation during sleep, while late negative dreams (occurring later in sleep) may indicate a failure in this mood regulation process [66]. To evaluate the influence of anxiety, possibly linked to depression, the STAI-Y questionnaire was administered, but it did not reveal significant score changes between the MOP and placebo groups at any time point. The significant reduction in REM sleep time could be attributed to the significant increase in SWS: since no significant differences were observed in the total amount of sleep, the increase in SWS occurred at the expense of REM sleep, which decreased.

In 2022, Benz et al. conducted a study comparing subjective and objective measures of sleep duration in a group of insomniacs and a group of good sleepers, finding large systematic differences between subjective and objective measures of sleep duration, with differing discrepancies in the two groups [68]. While 79% of insomniacs underestimated total sleep and 21% overestimated it, the opposite occurred in the good sleeper group, with 59% overestimating sleep duration and 41% underestimating it. Based on these results, the authors argue that subjective sleep measurements cannot be considered valid, as they do not align with objective measures, nor can objective sleep assessments be deemed valid, as they do not reflect the subjective experience. Benz et al. thus suggest that studies evaluating sleep duration should use both subjective and objective measures, as they are complementary and insufficient alone to establish such a complex concept as sleep quality [68]. Based on the suggestions of these authors and others, this study evaluated both objective parameters related to sleep duration and phase distribution using Garmin watches and subjective parameters through the combination of the above questionnaires. The subjective improvement in sleep was assessed using the CGI-I scale, and while 87% of subjects reported various degrees of improvement (slightly improved, greatly improved, significantly improved) during the MOP intake period, only 30% of the placebo group reported improvements, with a much lower degree than the supplemented group. In the placebo group, more than half of the participants reported no change in sleep quality (53%), and 17% even reported worsening compared to the initial situation. In contrast, in the MOP group, 67% of subjects reported slight improvement, 10% reported significant improvement, and another 7% saw very positive changes. Given the evidence that sleep quality has a strong subjective psychological component tied to the perception of sleep itself, the results of this study allow us to assert with a good degree of confidence the effectiveness of MOP.

The study’s cross-over design with a sample of 30 participants provided robust data, showing significant improvements in sleep quality and reductions in insomnia severity, as evidenced by ISI scores and enhanced deep sleep and REM phases. Furthermore, it also showed that the perceived improvement in sleep quality is statistically significant in the supplemented group. This is particularly important given that, as mentioned earlier, there are no universal and uniform criteria for evaluating sleep quality, so an improvement in a subjective assessment might reflect an actual improvement in sleep quality. Notably, the study emphasised that physical activity levels and anxiety did not significantly influence these outcomes, isolating the effects of the *Melissa officinalis* supplement. The Phytosome^®^ form proved particularly effective and safe, suggesting it as a viable alternative to traditional pharmaceutical treatments for insomnia, offering a natural and potentially safer option for individuals seeking relief from sleep disorders.

## 5. Conclusions

According to what has been recently published as regards to the effect played by MOP on GABA-T [69], our study demonstrated that MOP is effective in improving sleep quality and reducing insomnia severity in participants. The results, based on a cross-over design with 30 participants, showed significant improvements in Insomnia Severity Index scores and an increase in deep sleep (at the expense of the REM phase, without significant influence from physical activity or anxiety levels. The use of the STAI-Y questionnaire confirmed that anxiety did not alter the results, allowing the improvements to be attributed directly to the intake of MOP. Additionally, participants reported a subjective improvement in sleep quality, suggesting that the perception of sleep was positively influenced by MOP supplementation. These findings suggest that MOP may represent a natural and safe alternative to traditional pharmacological treatments for insomnia, and the fact that this study has been conducted with a rigorous methodological design, including the use of a placebo group, we have stable and reliable results because the inclusion of the placebo group allows for more accurate attribution of the observed improvements to the intervention, minimising potential confounding factors. However, future studies could further explore the role of deep sleep in improving cognitive and memory functions while also assessing the long-term effects of phytosome intake. Moreover, it would be useful to expand the study sample and conduct research on diverse populations to confirm and generalise the findings, also exploring potential interactions with other sleep disorders or psychological conditions.

## Figures and Tables

**Table 1:** Features of the eligible subjects (*n* = 30) who participated in the trial (all of them completed the study) over a one-month period, from March to April, in 2024. *χ*^2^: chi-square test; t: t value; *p*: *p* value for the relative test; BMI: body mass index.

		Males	Fe Femalesmales	
		*n* (%) 13 (43%)	*n* (%) 17 (57%)	
Education	Middle School	2 (15%)	0 (0%)	
	High School	5 (38%)	11 (65%)	*χ*^2^ (*p*) = 3.78 (0.151)
	Degree	6 (46%)	6 (35%)	
		Mean ± SD	Mean ± SD	*t* (*p*)
Age (years)		45.2 ± 11.6	45.3 ± 12.3	−0.019 (0.985)
Weight (kg)		73.5 ± 13.8	72.5 ± 13.2	−2.990 (0.006)
Height (cm)		173.2 ± 7.6	171.4 ± 8.9	−4.301 (0.0002)
BMI (kg/m^2^)		24.0 ± 3.5	23.1 ± 2.7	−0.985 (0.333)

**Table 2:** Subjective perception of sleep quality. MOP: *Melissa officinalis* phytosome; P: placebo.

.	MOP	P
Sightly worsened	3% (1)	17% (5)
No change	13% (4)	53% (16)
Sightly improved	67% (20)	17% (5)
Greatly improved	10% (3)	13% (4)
Significantly improved	7% (2)	0% (0)

## Data Availability

The raw data supporting the conclusions of this article will be made available by the authors on request due to ethical reason.
